# Putting the pieces together: Clinical judgment from the perspective of registered nurse anaesthetists and anaesthesiologists at tracheal extubations after general anaesthesia

**DOI:** 10.1186/s12871-026-04143-w

**Published:** 2026-08-12

**Authors:** Linda Rönnberg, Emma Brulin, Bodil J. Landstad, Christina Melin-Johansson, Ulrica Nilsson, Maria Härgestam

**Affiliations:** 1 Department of Nursing, Faculty of Medicine, Umeå University, Umeå, Sweden; 2 Department of Health Sciences, Division of Nursing, Mid Sweden University, Östersund, Sweden; 3 Perioperative unit, Region Jämtland Härjedalen, Östersund Hospital, Östersund, Sweden; 4 Institute of Environmental Medicine, Karolinska Institutet, Stockholm, Sweden; 5 Department of Health Sciences, Division of Public Health Science, Mid Sweden University, Östersund, Sweden; 6 Department of Public Health and Clinical Medicine, Umeå University, Umeå, Sweden; 7 Unit of Research, Östersund Hospital, Region Jämtland Härjedalen, Östersund, Sweden; 8 Department of Neurobiology, Care Sciences and Society, Karolinska Institutet, Stockholm, Sweden

**Keywords:** Anaesthesia setting, Anaesthesiologists, Clinical judgment, Interdisciplinary teamwork, Registered nurse anaesthetist, Reflexive thematic analysis, Tracheal extubation, Triangulation

## Abstract

**Background:**

Extubation of patients undergoing general anaesthesia is a shared concern among registered nurse anaesthetists and anaesthesiologists. Although it has been described as complex and unpredictable, knowledge of this critical moment remains limited. Clinical judgment is consistently applied when making crucial decisions regarding patient care. This study aimed to investigate how RNAs and ANs expressed clinical judgment during extubation through noticing, interpreting, responding, and reflecting.

**Methods:**

A qualitative reflexive thematic analysis was conducted, using interviews with registered nurse anaesthetists and anaesthesiologists from university and regional hospitals in Sweden. Triangulation was used to explore the perspectives of registered nurse anaesthetists (*n* = 20) and anaesthesiologists (*n* = 17) at extubations. Two datasets, one from focus group interviews with registered nurse anaesthetists and one from individual interviews with anaesthesiologists, were used to triangulate data.

**Results:**

Extubation was described as a dynamic process of clinical judgment, where anaesthesia professionals continuously integrated clinical cues, experience, and situational awareness to ensure patient safety. Multisensory patient-related and situational cues were attended to and integrated to form an initial understanding of the patient’s condition. These cues were interpreted through prior experience and pattern recognition, while responses were shaped by context and often guided by embodied intuition. Reflection both in action and on action supported ongoing adjustment and the development of clinical judgment over time.

**Conclusions:**

During extubation, registered nurse anaesthetists and anaesthesiologists exercised clinical judgment by putting the pieces together, continuously integrating clinical cues, experience, and situational awareness. This process involved multisensory noticing, experience-based interpretation, context-dependent responding often guided by embodied intuition, and ongoing reflection to ensure patient safety. These findings highlight the importance of supporting clinical judgment through experience-based learning, reflective practice, and work environments that enable focused attention during extubation.

## Background

Clinical judgment (CJ) is the process consistently applied in patient care decision-making [1]. It is a reflective, knowledge-based reasoning process guided by comprehensive knowledge [2] that requires an understanding of situations, assessment of patient concerns, and responsiveness to patient needs [3]. It is fundamental to interpreting patient information and delivering effective care that directly influences patient outcomes [4]. In the anaesthesia setting, CJ is particularly relevant for decision-making during the complex and unpredictable process of extubation.

Tanner [5] developed a research-based CJ model (CJM) to illustrate the application of clinical reasoning and decision-making in the complexity of patient care, including changing conditions, adaptive strategies, and the selection of appropriate actions. In the CJM context, background and relationship interact and influence one another. Information from these components is integrated to support decision-making and high-quality, patient-centred care [5]. The CJM consists of four stages: noticing, interpreting, responding, and reflecting. *Noticing* refers to perceiving salient aspects of the situation. *Interpreting* involves making sense of what is noticed and determining an appropriate course of action. *Responding* entails acting while beginning the first phase of *reflecting*, i.e., evaluating both the action itself and its outcome. *Reflecting* occurs both in and on action, enabling professionals to make sense of and learn from experience. Together, these stages constitute the enactment of CJ in practice and can guide our understanding of how ANs and RNAs make decisions about extubation.

This study focuses on endotracheal extubations performed in the operating room (OR). In Sweden, extubations in the OR are commonly performed by RNAs or ANs, who are responsible for providing safe anaesthesia care throughout the perioperative period [6]. At the end of the anaesthesia period, the patient is vulnerable and requires a high level of precision from RNAs and ANs. While monitoring data support decision-making, both RNAs [7] and ANs [8] also rely on their experience and situational awareness to ensure patient safety. Extubation should be carefully considered on an individual basis for each patient and surgical procedure [9] as a substantial proportion of severe perioperative adverse outcomes, such as death and brain damage, occur during the extubation phase [10]. Planned extubation is an elective process that requires careful assessment of risk factors, patient optimisation, and thorough preparation, including ensuring that appropriate equipment is immediately available and that clinicians are skilled in its use [10]; however, despite this structured approach, extubation decisions are often still guided by intuition or “gut feelings” that can be difficult to define clearly [7, 8]. While CJ develops through experience, clinical practice, theoretical knowledge, and critical analysis [1], inexperienced RNAs and ANs may therefore find extubating challenging. This poses challenges to the transfer of learning to less-experienced RNAs and ANs. To gain a deeper understanding of the tacit, experience-based aspects of CJ, this study investigated how RNAs and ANs expressed CJ during extubation through noticing, interpreting, responding, and reflecting.

## Method

This study uses a qualitative design, triangulating interview data from RNAs and ANs working in anaesthesia units at university and regional hospitals in Sweden. Reporting of this study was guided by the Consolidated Criteria for Reporting Qualitative Research (COREQ) checklist for interviews and focus groups. The checklist was used to enhance transparency in reporting study design, data collection, and analysis [11].

### Study design

The interview material derives from interviews with a total of 37 RNAs and ANs. Participants in the original studies were recruited using consecutive sampling from participating anaesthesia departments [7, 8]. Information about the studies was provided face-to-face and via e-mail by the first author. Prior to the interviews, the participants were asked to reflect on three types of extubations they had experienced: a normal, a good, and a not-so-good situation. During the interviews, participants were invited to describe and discuss these experiences using the same pilot-tested semi-structured interview guide in both studies.

First, we conducted six focus group interviews with 20 RNAs from two hospitals (15 women, 5 men) between February and April 2014. Eleven participants were from a university hospital and nine from a regional hospital. Their professional experience ranged from 1.5 to 24 years. Ages ranged from 30 to 63 years. The focus group interviews were conducted to facilitate discussion around shared experiences, teamwork, and interaction during extubation.

Second, 17 individual interviews with ANs and anaesthesia residents (all referred to as ANs in this study) from three regional hospitals (5 women and 12 men) were conducted between August and September 2017. Professional experience as ANs varied from 1 year to 40 years. Ages ranged from 32 to 64 years. Individual interviews were conducted with ANs to enable more in-depth exploration of individual clinical reasoning and decision-making processes during extubations.

In both studies, sample adequacy was guided by the concept of information power, where the specificity of the aim, the relevance of the sample, and the richness of the data supported the sample size [12]. All interviews were conducted and audio-recorded by the first author at the participants’ workplaces. Each participant took part in one interview, and no participants withdrew from either study. CM-J and UN served as observers during the focus group interviews at one hospital each. Participant checking was not conducted. At the time of data collection, the first author was an RNA and PhD student.

### Data analysis

The data were analysed using reflexive thematic analysis (RTA) [13] in combination with methodological triangulation (MT) [14]. MT involves analysing data through multiple perspectives and theoretical lenses to enhance depth and interpretative richness. The combination of RTA and MT was used to gain an in-depth understanding of how CJ was expressed during extubations and to identify potential similarities and differences between the professions (RNA and AN).

Tanner’s CJM [5] constituted the theoretical lens in the triangulation. The model’s four phases, noticing, interpreting, responding and reflecting, were used as overarching analytical domains. The analysis followed an iterative and reflexive process, with coding and interpretation of participants’ accounts conducted inductively and continually refined in dialogue with the data. Although informed by Tanner’s model, themes were generated from patterns of meaning grounded in participants’ descriptions rather than from predefined categories.

The analysis began with repeated readings of the transcripts to achieve familiarity with the data, identify patterns and gain a broader understanding of the content [15]. Initial codes were generated through repeated engagement with the transcripts and were subsequently clustered into broader interpretive themes. Codes were generated by identifying relevant concepts and phrases in line with the study aim [15]. To enhance credibility [16], MH read three transcripts from both the focus groups and the individual interviews and discussed emerging interpretations and patterns with LR. Throughout the analysis, LR, MH, BJL, EB, and UN discussed the codes and emerging themes, returning repeatedly to the transcripts to critically examine and refine the developing interpretations. After initial clustering of codes, themes were created. The authors regularly met to discuss the analysis and compare findings against Tanner’s CJM and prior research. Reflexivity was maintained through ongoing discussions among the authors regarding assumptions, interpretations, and alternative understandings of the data. To contextualise and illustrate the RNAs’ and ANs’ descriptions, verbatim participant excerpts are inserted in the results. Professional affiliation (RNA or AN) was retained throughout the coding and interpretation process to preserve both similarities and differences during the integrated analysis.

## Results

The results comprise one overarching theme: Putting the pieces together. Eight themes show how RNAs and ANs expressed CJ during extubation through noticing, interpreting, responding, and reflecting (Table 1).

**Table 1:** An overview of the included overarching theme and themes

Overarching theme	Putting the pieces together			
Tanner’s Model of Clinical Judgment	Noticing	Interpreting	Responding	Reflecting
Themes	Making sense of the patient through multisensory cues	Constructing meaning from clinical cues	Acting through embodied intuition in dynamic situations	Ensuring patient safety through reflection in action
	Recognising situational cues in the clinical context	Drawing on experience to guide clinical reasoning	Responding in context: the work environment shaping extubation practice	Developing clinical judgment through reflection and experience

### Putting the pieces together

**Fig. 1:**
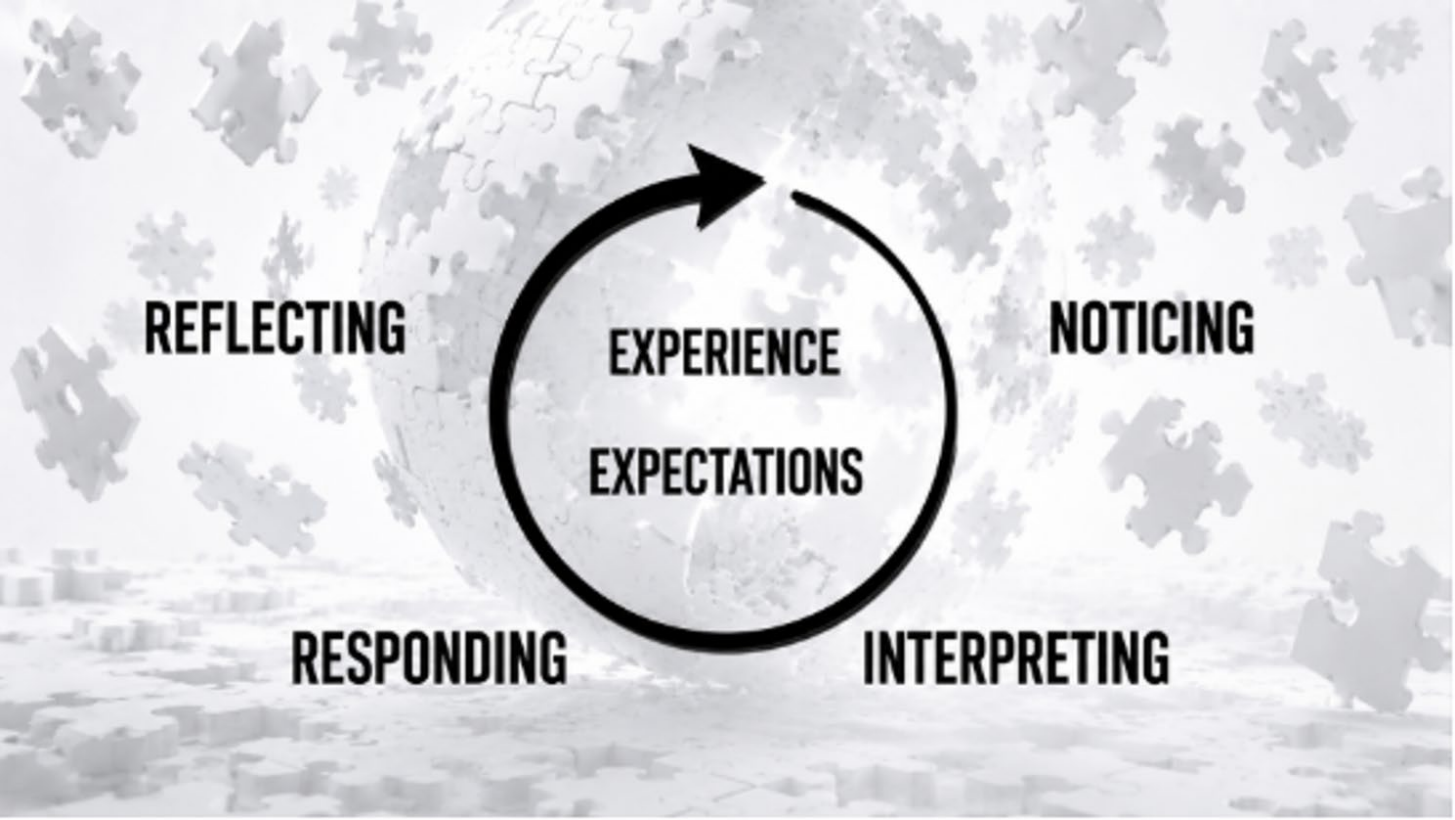
RNAs and ANs integrate expectations, patient observations, and clinical measurements to assemble a coherent understanding of the patient’s readiness for extubation. The puzzle design illustrates how multiple pieces of clinical information are continuously integrated and reconfigured throughout the iterative and dynamic process of clinical judgment during extubation. Reflection may influence future noticing, interpretation, and responses as clinicians continuously adapt their understanding according to the patient’s changing condition, prior experiences, and the clinical context

The overarching theme, *Putting the pieces together*, illustrates that decisions about extubation are not made instantly; they are a process in which RNAs and ANs assemble pieces through noticing, interpreting, responding, and reflecting (Fig. 1). Pieces are collected by assembling cues and staying attentive and present with the patient. Some pieces they bring to the situation involve their expectations for extubation, based on experience, given the unique patient. This is a non-linear, dynamic, iterative process that evolves over time in which they monitor vital signs, observe the patient’s responses, and identify cues that may indicate potential extubation difficulties. The reflection feeds back into future noticing, interpretation, and responses.

The RNAs and ANs use CJ to assess extubation readiness, forming a coherent view that guides action. As the process unfolds, more pieces are gathered, and they put them together to create a clearer picture on which they can base their decision to extubate. Sometimes, pieces are missing, and it may not be easy to see the entire picture of the extubation. When reflecting on extubations, pieces are gathered to build experience and create expectations for future extubations.

Experiences are central to how RNAs and ANs express CJ and to the analytical dimension of how they express noticing, interpreting, responding, and reflecting. Experiences shape the expectations that RNAs and ANs have regarding extubation.

### Noticing

During the extubation process, RNAs and ANs similarly expressed Noticing through the themes *Making sense of the patient through multisensory cues* and *Recognising situational cues in the clinical context.* Noticing involved attending to multisensory patient reactions as cues, which were taken up in relation to contextual factors and prior experience. These cues were engaged in an ongoing patterning process, in which earlier experiences of extubation informed what was attended to and how changes in the patient were noticed. Noticing unfolded over time, beginning during the pre-anaesthesia assessment and continuing throughout the perioperative phase. As the situation developed, participants described how patient reactions served as cues that contributed to a gradually forming sense of the upcoming extubation.

#### Making sense of the patient through multisensory cues

The RNAs and ANs described planning for extubation as beginning during the pre-anaesthesia assessment and continuing as a dynamic, evolving process shaped by ongoing changes in patient condition. During this phase, patient-related cues such as anatomy, medical history, and potential airway difficulties were assessed, contributing to an initial understanding of the upcoming extubation. This understanding was further developed by attending to multisensory cues, including physiological measurements and bodily signs. Based on these cues, participants described forming a mental preparation and an initial picture of the extubation. This picture continued to evolve throughout the anaesthesia period through ongoing clinical observations and continuous reassessment of the patient’s condition. One AN described the importance of physically managing the extubation procedure:

“When I am called in for extubation, I prefer to remove the tube myself, so you feel it in your hands and feel how the patient responds” (AN.22).

The RNAs and ANs described maintaining vigilance and remaining attentive to subtle patient changes throughout the extubation process. Staying focused and physically close to the patient was considered essential for detecting cues, signs of stress, awakening, and readiness for extubation. RNAs were particularly described as rarely leaving the patient’s side during this phase, whereas ANs emphasised the importance of being present during extubation. As extubation approached, participants described becoming increasingly alert to signs such as changes in heart rate, blood pressure, respiratory frequency, and patient awareness. Observing the patient’s eyes was repeatedly described as an important way of assessing consciousness and determining readiness for extubation. One AN explained:

“Their eyes mirror their awareness; I know what to look for before extubation.” (AN.29).

Both RNAs and ANs described how noticing patient reactions and physiological changes continuously shaped their understanding of the situation and guided their actions during extubation.

#### Recognising situational cues in the clinical context

This was developed as the second theme of noticing. RNAs and ANs described how they recognised situational cues in the clinical context, such as the type of surgery, anaesthetic management, and phase of the procedure, and how these cues contributed to the formation of expectations for extubation. Participants described drawing on experiences from previous, similar situations to recognise patterns, identify potential risks, and anticipate how the extubation might unfold. Prior difficult extubations were described as particularly influential, contributing to increased attentiveness and preparedness in subsequent situations. One RNA explained:

“After experiencing an extubation that didn’t go well, you become more alert. You recognise situations and are more careful next time.” (RNA.12).

Experience was described as central to recognising subtle changes in patients and understanding how different clinical situations could develop during extubation. Less experienced RNAs and ANs described relying more on theoretical knowledge, structured assessments, and support from colleagues. They emphasised the importance of having assistance available and learning through observation of more experienced colleagues. Through repeated clinical exposure and reflection on previous situations, participants gradually developed confidence in recognising complex extubation situations and became increasingly attentive to cues and subtle clinical changes. Despite differences in professional roles, both groups described similar processes of learning to recognise patterns in relation to situational cues during extubation.

### Interpreting

Interpretation was expressed through the themes *Constructing meaning from clinical cues* and *Drawing on experience in clinical reasoning*. Building on what had been noticed, RNAs and ANs described engaging with physiological responses while monitoring data and situational information. As extubation progressed, these sources of information were brought together in an ongoing process through which observations took on clinical meaning. This developing sense of the patient’s condition informed decisions about when and how extubation was performed.

#### Constructing meaning from clinical cues

RNAs and ANs similarly described how they constructed meaning from patient reactions and physiological changes by integrating observations, clinical reasoning, and prior experience. Through ongoing interpretation of what had been noticed, they made sense of the patient’s condition in relation to the upcoming extubation. They also described integrating information from monitors, medical records, bodily signs, and communication within the OR team. Clinical reasoning involved synthesising multiple sources of information into an overall understanding of the patient’s condition. One RNA described this process as follows:

“I see it, and I feel it. I have it in my hands; it is all the clinical experience I gathered. I mentally know when they are ready for it [the extubation], we are connected.” (RNA.10).

Another RNA in the focus group continued:

“You put it all together, all the things you gathered, you put into your decision.” (RNA.9).

RNAs and ANs described interpreting observations in relation to the individual patient and continuously reassessing whether normal respiration, circulation, airway reflexes, and neuromuscular function had sufficiently returned. The interpretation process involved linking separate observations into an increasingly coherent understanding of the extubation situation. This meaning-making process involved continuously relating new clinical cues to prior expectations and patterns, integrating them into an evolving understanding of the patient’s condition.

#### Drawing on experience to guide clinical reasoning

Experience emerged as central to clinical reasoning during extubation. RNAs and ANs similarly described how clinical cues such as pupil responses, breathing patterns, skin colour, body movements, and subtle physiological changes were interpreted through accumulated clinical experience. Participants emphasised that experience shaped how they attended to and interpreted both monitor values and patient reactions. One AN described how their focus shifted over time from primarily relying on monitors to integrating multiple clinical cues more holistically:

“In the beginning, I only looked at the monitors. I was afraid of missing something, but now I know which patient to keep an eye on. I mean, the monitor with its values is important, but it is a complement to clinical skills.” (AN.30).

Experience was associated with increased confidence, pattern recognition, and a more intuitive way of interpreting extubation situations. In contrast, those with less experience described greater uncertainty in interpreting patient reactions and in understanding the atmosphere in the OR during stressful situations. One AN explained:

“I can see how the patient struggles, and I can feel it, the atmosphere in the OR if they [colleagues] are stressed when I come in, but still, I cannot really, not nearly understand what it stands for. I need more practice in this kind of situation, and I need to be more in the centre of action.” (AN.23).

The participants described how repeated exposure to extubation situations gradually strengthened their ability to recognise patterns and anticipate how situations would develop. Experienced RNAs and ANs described “knowing” when extubation could proceed safely, whereas inexperienced clinicians relied more heavily on theoretical knowledge, structured assessments, and support from colleagues.

Subtle clinical signs were described as particularly important during interpretation. Participants referred to observing small changes in capnography curves, eye signs, breathing patterns, and body movements when assessing the patient’s readiness for extubation. One AN summarised this process:

“I gathered the signs I knew would come after reducing the anaesthetic drugs and starting to wake them [the patient]; it is individual, but I know what to look for.” (AN.26).

The RNAs and ANs described clinical reasoning during extubation as a process in which previous experiences, physiological observations, and situational awareness were continuously integrated to guide interpretation and subsequent actions.

### Responding

Drawing on their understanding of the patient’s condition, RNAs and ANs described determining how and when extubation should proceed, often under conditions requiring rapid decision-making and sustained attention. The RNAs and ANs thus responded to clinical cues and adapted their actions based on what they had noticed and interpreted. Responding was expressed through the themes *Acting through embodied intuition in dynamic situations* and *Responding in context: the work environment shaping extubation practice*. While both professions emphasised these demands, differences emerged in how responsibility, teamwork, and support were experienced and enacted during the extubation phase.

#### Acting through embodied intuition in dynamic situations

Intuition emerged as central during responding. Both RNAs and ANs described how rapid changes in the patient’s condition often required immediate actions grounded in experience, embodied knowledge, and gut feeling. Intuitive responses were described as developing through repeated exposure to similar extubation situations and becoming integrated with clinical cues, physiological observations, and monitor data.

The RNAs and ANs described acting on subtle changes in the patient’s condition that were sometimes difficult to fully articulate. These responses were not perceived as separate from clinical knowledge but as grounded in prior experience, situational awareness, and recognition of familiar patterns during extubation. One AN described responding intuitively during a sudden deterioration in the patient’s condition:

“Then, when I saw how he suddenly lost blood pressure, I used the syringe that I had in my hand already; I kind of already knew somehow.” (AN.23).

The RNAs and ANs also described preparing medications and equipment in advance based on expectations of how the extubation situation might develop. In this way, intuition was closely connected to preparedness, anticipation, and the ability to rapidly recognise and act in situations that could become unsafe.

The RNAs and ANs explained that gut feelings often directed their attention to situations that “did not feel right,” sometimes before measurable changes became fully apparent. Through experience, they developed confidence in acting rapidly on subtle clinical cues while simultaneously integrating monitor data, physiological signs, and clinical observations.

Responding during extubation was thus described as a dynamic process in which intuition, clinical cues, and situational awareness were continuously integrated to guide immediate actions in a rapidly evolving clinical context. Intuition thus functioned as an embodied form of CJ, enabling rapid responses in situations where time for deliberation was limited.

#### Responding in context: the work environment shaping extubation practice

RNAs and ANs described how responding during extubation was shaped not only by clinical cues but also by the OR work environment. Participants described relying on both clinical observations and physiological measurements when responding to the patient’s condition, while simultaneously attending to contextual factors within the OR.

Participants emphasised the importance of relating the patient’s condition to the surrounding environment, particularly during the vulnerable awakening phase. Although similarities in clinical judgment were prominent across professional groups, differences emerged in how clinical judgment was enacted during extubation. These differences appeared to be less related to fundamentally different clinical judgment processes and more related to professional responsibilities, training backgrounds, and organisational roles. Despite these differences, both professions engaged in similar iterative processes of noticing, interpreting, responding, and reflecting. However, differences between professional roles became apparent in how the work environment was experienced.

RNAs more frequently described disturbances and interruptions during extubation. They characterised the situation as vulnerable and associated with pressure, limited communication, and competing demands. Several RNAs reported feeling isolated despite the presence of other professionals and described situations in which they felt pressured to proceed more quickly than they considered appropriate. One RNA described the need for calm and minimal disturbance during extubation:

“Then, at extubations, it should be like at the intubation, peaceful, not anyone making noise, touching the patient, or talking to me. I mean, it affects both the patient and me. I mean, I can be stressed by not having time to wait for the right time to extubate.” (RNA.9).

Participants described how noise, conversations, and ongoing activity in the OR could disturb both the patient and the clinicians’ concentration during emergence from anaesthesia. RNAs explained that such interruptions could affect their ability to maintain focus, shaping how and when they acted during extubation. One RNA explained:

“When you’re standing there, ready to wake the patient, it’s as if they forget about the patient. They’re finished, but the patient and I, well, we still have a tube to remove. It’s distracting, but there’s not much I can do because then I would be causing a disturbance too.” (RNA.8).

In contrast, ANs more often described feeling integrated within the OR team and experiencing greater influence over the environment during extubation. Several ANs explained how their presence shaped the atmosphere in the OR and how they were expected to assume responsibility for extubation upon entering the room. At the same time, they described balancing responsibility for patient safety while not always being continuously present at the bedside.

Participants from both professions emphasised the importance of maintaining focused attention and minimising unnecessary disturbances as the patient emerged from anaesthesia.

### Reflecting

Reflecting was expressed through ongoing and retrospective engagement with extubation experiences. In this study, reflecting refers to how RNAs and ANs continuously evaluated and reassessed their actions during extubation (reflection-in-action) and revisited their experiences afterwards (reflection-on-action) to support learning and future CJ. Reflecting was expressed through the themes *Ensuring patient safety through reflection in action* and *Developing clinical judgment through reflection and experience*. While extubation was described in similar ways across professions, differences emerged regarding responsibility, perceived challenges, and opportunities for reflection.

#### Ensuring patient safety through reflection in action

RNAs and ANs described how ensuring patient safety during extubation involved continuous reflection-in-action, in which they monitored, evaluated, and adjusted their responses in relation to the patient’s condition and the surrounding work environment. Rather than acting on cues alone, participants described an ongoing awareness of how their actions influenced the patient and the extubation process.

Participants reflected on how both clinical cues and contextual factors within the OR shaped their ability to maintain patient safety, particularly during the vulnerable awakening phase. This involved continuously reassessing the situation and adapting their responses in relation to changing patient conditions and environmental demands.

RNAs more frequently reflected on the impact of disturbances and interruptions during extubation. They described how noise, limited communication, and competing demands could challenge their ability to maintain focus and enact safe care. Several RNAs reflected on situations where they felt pressured to proceed more quickly than they considered appropriate, highlighting tensions between CJ and environmental constraints.

Participants described how ongoing activity in the OR required them to remain attentive not only to the patient but also to the ways environmental factors influenced their concentration and actions. RNAs reflected that interruptions could necessitate increased vigilance and conscious effort to maintain control over the situation. In contrast, ANs more often reflected on their role within the OR team and how this influenced the management of extubation. They described being able to shape the environment and balance responsibility for patient safety while relying on collaboration with the team. At the same time, they reflected on the challenges of not always being continuously present at the bedside.

One AN described this responsibility as follows:

“I have to trust the nurses. Sometimes, I do not know until they call me, and it has become urgent. But often, when I come in, all is peaceful and prepared.” (AN.26).

Across both professions, participants emphasised the importance of maintaining focused attention and minimising unnecessary disturbances as the patient emerged from anaesthesia. Reflection in action was described as a way of continuously adjusting behaviour to protect patient safety within a dynamic and sometimes unpredictable clinical environment.

#### Developing clinical judgment through reflection and experience

RNAs and ANs described how reflection after extubation situations contributed to learning and the development of CJ over time. Participants described revisiting previous extubations, particularly those that were challenging or associated with uncertainty, in order to better understand what had occurred and how similar situations might be managed in the future.

Reflection-on-action was described as a way of critically appraising one’s own clinical reasoning and responses. Participants described how they evaluated decisions, timing, and actions taken during extubation, often in relation to patient outcomes and the overall course of events.

Challenging or adverse extubation situations were highlighted as particularly important for learning. Participants described how such experiences prompted deeper reflection and led to changes in their approach to subsequent extubations. Through this process, they developed greater awareness of subtle clinical cues, timing, and situational factors influencing patient safety.

Learning was also described as occurring through informal discussions with colleagues, where experiences were shared and reflected upon within the team. These reflections contributed to a collective understanding of extubation practice and supported the development of both individual and shared clinical knowledge.

Over time, participants described how reflection contributed to increased confidence and a more refined CJ. Experiences were internalised and transformed into practical knowledge, influencing how future extubation situations were understood and managed.

Reflection-on-action was thus described as an essential component in the ongoing development of CJ, linking past experiences to future practice and strengthening the ability to respond safely in complex extubation situations.

## Discussion

Drawing on interview data from 37 RNAs and ANs, the results of this study illustrate that extubation is a dynamic, experience-based process highly dependent on the clinical situation, in which RNAs and ANs continuously engage with patient cues while providing care. Tanner’s [5] CJM provided a useful framework for understanding how noticing, interpreting, responding, and reflecting were enacted in this context. The overarching theme, *Putting the pieces together*, captures how CJ during extubation unfolds as a continuous process in which patient reactions, physiological measurements, and situational conditions are integrated and shaped by prior experience. As illustrated, this process is best understood as cyclical rather than linear, where noticing, interpreting, responding, and reflecting are interconnected and continuously influenced by experience and expectations. Within this process, noticing involves attending to patient cues; interpreting entails constructing meaning by relating these cues to physiological and contextual information; responding refers to acting in dynamic situations; and reflecting involves revisiting and reshaping understanding for future practice.

Our findings illustrate that the phases of CJ are not discrete or sequential but continuously interconnected, forming a recursive process shaped by both experience and context. Thus, *putting the pieces together* represents not only an ongoing clinical process, but also the gradual development of professional competence over time. This is consistent with previous research emphasising how professional competence develops through the integration of experience, reflection, and situated clinical practice [17]. Accordingly, CJ in extubation may be understood as an evolving, experience-based process in which knowing, doing, and reflecting are inseparably intertwined in the ongoing pursuit of patient safety.

The importance of anticipatory assessment is supported by previous research emphasising the role of pre-anaesthetic evaluation in identifying potential risks and preparing for extubation [9, 18]. In line with our findings, this suggests that CJ begins prior to the extubation phase, as clinicians form expectations and prepare for potential challenges.

Although the metaphor of a puzzle suggests the assembly of discrete pieces of information, participants did not describe extubation as resulting in a single, fixed, or fully predictable outcome. Rather, extubation was characterised as a continuously evolving process marked by uncertainty, adaptation, and ongoing revision. In this sense, the “puzzle” is never fully completed but continuously reconfigured as patient conditions change and clinicians’ experience develops. This interpretation aligns with Tanner’s [5] CJM. However, CJ during extubation was not only iterative but also explicitly experience-driven, embodied, and contextually situated. Participants described moving between noticing patient reactions as clinical cues, constructing meaning, responding to changes, and reflecting both during and after extubation. Reflection further influenced subsequent noticing and interpretation, indicating that CJ develops over time through repeated exposure and accumulated experiential knowledge.

The findings further indicate that learning and the development of CJ are closely embedded within everyday extubation practice. Participants described how repeated exposure to extubation situations, combined with reflection and collaboration, supported their ability to recognise patterns and attend to multisensory cues. This aligns with previous research highlighting the importance of multisensory observation in anaesthesia practice, particularly when patients are unable to communicate verbally and where early detection of physiological changes is essential for patient safety [19, 20]. This underscores the importance of workplace learning in shaping clinical competence. As Billett [21] suggests, professional learning is grounded in participation in authentic activities, interaction with colleagues, observation, and reflection. Differences in experience levels were evident, with less experienced clinicians relying more on structured assessments and external support, whereas more experienced practitioners demonstrated greater fluency in recognising patterns and integrating cues into their CJ. These findings are consistent with research demonstrating that learning in clinical practice differs between novices and experienced clinicians, with experience-based knowledge and organisational memory playing a central role [22, 23]. Similarly, Larsson and Holmström [24] highlight that the development of expertise in anaesthesiology depends on engagement in complex situations combined with reflection and collaboration with more experienced colleagues.

A central finding of this study concerns the role of embodied intuition during responding, suggesting that responding is not solely based on deliberate reasoning but involves an integration of analytical and intuitive processes. This interpretation aligns with previous research describing intuition as an integral component of CJ and expert practice, emerging from experience and guiding action in complex situations [1, 5]. Participants described how rapid decision-making was often guided by subtle feelings or bodily sensations, particularly in situations where physiological data alone were insufficient. Importantly, intuition was not described as separate from clinical reasoning but as grounded in prior experience, situational awareness, and recognition of familiar patterns. Similar findings have been reported by Robben et al. [25], who describe how intuitive awareness supports action in complex and rapidly changing clinical situations. Such intuitive processes have also been described as forms of tacit, experience-based knowledge, where clinicians recognise patterns and act rapidly in familiar situations [25, 26]. The RNAs and ANs also described a shift with increasing experience, from relying primarily on monitor data to engaging more directly with the patient. Observations such as breathing patterns, eye signs, muscle tone, and subtle behavioural changes were central to interpreting the patient’s condition during emergence from anaesthesia. These findings reinforce the understanding of CJ as situated and embodied, rather than solely dependent on measurable parameters [5, 23]. They also align with findings from Jansson et al. [17], who describe how situational awareness develops through repeated engagement in clinical practice and is closely linked to the development of tacit knowledge and trust in one’s own competence.

Although similarities in clinical judgment were prominent across professional groups, differences emerged in how clinical judgment was enacted during extubation. These differences appeared to be less related to fundamentally different clinical judgment processes and more related to professional responsibilities, training backgrounds, and organisational roles. RNAs described a continuous bedside presence with ongoing sensory assessment and immediate adjustments, whereas ANs more often described broader risk assessment and supervisory decision-making. Despite these differences, both professions engaged in similar iterative processes of noticing, interpreting, responding, and reflecting. Differences between RNAs and ANs were particularly evident during the responding and reflecting phases. While both professions described extubation as requiring rapid decision-making and sustained attention, RNAs more frequently reported experiences of vulnerability, interruptions, and a sense of isolation. They typically remained continuously present at the bedside, balancing patient safety with workflow demands and time pressure. In contrast, ANs more often described supervisory roles and a stronger sense of integration within the OR team. Their presence was perceived as influencing team dynamics and carrying an expectation of responsibility when entering the room during extubation. These differences in roles and team integration appear to shape how extubation is both managed and experienced, with potential implications for CJ, clinician experience, and patient safety. RNAs described how interruptions, conversations, and preparation for subsequent patients could disrupt concentration during emergence from anaesthesia, thereby influencing how they maintained focus and enacted clinical responses. Similar challenges have been reported in anaesthesia and perioperative practice, where distractions, multitasking, and workflow interruptions may adversely affect performance and patient safety [27, 28]. Evidence from other high-risk clinical environments similarly indicates that frequent interruptions can compromise patient safety by increasing cognitive load and reducing clinicians’ ability to maintain situational awareness [29, 30]. Maintaining focused attention during extubation may therefore be critical for safe care. Our findings further indicate that disturbances during extubation may compromise both concentration and perceived safety, particularly for clinicians continuously engaged at the bedside. Participants described how environmental noise, competing demands, and limited communication could affect their ability to maintain situational awareness during this vulnerable phase. These findings extend previous research by illustrating how such disturbances are experienced in real-time clinical practice and how they influence clinicians’ ability to enact safe care. Communication and teamwork are widely recognised as essential for safe extubation [10]. In line with this, the present findings suggest that structured communication may support shared understanding, reduce uncertainty, and strengthen teamwork during extubation. For example, brief pre-extubation discussions clarifying roles, expectations, and anticipated responses could enhance coordination and patient safety during emergence from anaesthesia. Finally, reflection emerged as a central mechanism linking experience to the ongoing development of CJ. Participants described engaging in reflection both during extubation and retrospectively following challenging situations. This allowed them to revisit actions, identify patterns, and develop preparedness for future scenarios. Reflection thus supported both immediate adjustments in practice and longer-term learning. The present findings contribute to existing knowledge by demonstrating how reflection operates across both reflection-in-action and reflection-on-action, supporting the continuous development of CJ in complex situations. This is consistent with Tanner’s model [5], in which understanding evolves as the reasoning process unfolds. Rapid recognition may lead to intuitive responses, whereas more complex situations require deliberate reflection. Less experienced clinicians particularly emphasised the importance of guidance, observation, and shared reflection with more experienced colleagues, supporting previous research identifying workplace learning as central to the development of clinical competence [21].

### Methodological discussion

The triangulation strategy, drawing on focus groups and individual interviews, was a methodological strength, enabling the phenomenon to be explored from multiple sources and perspectives [14, 31]. Including both RNAs and ANs supported validation through data source triangulation and enhanced the comprehensiveness and credibility of the findings. Triangulation among the authors further strengthened the interpretation of data through a critical discussion of emerging findings. Throughout the analysis, reflexivity was maintained through ongoing analytic discussions among the authors, with diverse clinical and academic perspectives contributing to challenging and refining the analytical interpretations.

A potential limitation is that the data were collected some years ago. Although the core cognitive processes of CJ are likely stable, clinical, technical, and organisational practices at the time of data collection may differ from current practices; this should be considered when interpreting the findings. Also, three authors were actively engaged in clinical anaesthesia practice during this project, and the research team contributed diverse clinical and academic perspectives to the analysis, thereby supporting contextual relevance and analytical dependability. These authors’ professional experience and prior understanding as RNAs may have influenced both data collection and interpretation. However, this background also facilitated rapport and understanding of the clinical context, enabling participants to speak openly about complex extubation situations. To address this, the analytical process involved ongoing reflexive discussions within the research team and a sustained effort to ground interpretations in participants’ accounts.

The choice of interview format was influenced by both methodological and practical considerations. Both professional groups were interviewed using the same interview guide and were asked to reflect on similar extubation experiences, supporting comparability across datasets. Focus group interviews were considered suitable for RNAs, as they enabled discussion of shared clinical experiences and interaction during extubation. Individual interviews were used for ANs to allow in-depth exploration of individual clinical reasoning. Talking about problematic extubation may be more difficult for ANs in a group setting, as concerns related to occupational stress stigma and differences in perceived social support may influence professionals’ willingness to discuss challenging clinical experiences [32, 33]. Importantly, because ANs were fewer in number, particularly at smaller hospitals, focus group interviews were difficult to arrange during working hours without compromising clinical staffing. Although differences in interview format may have influenced how experiences were articulated, we do not believe they fully account for the observed differences between professions; these more likely reflect differences in professional roles and responsibilities during extubation. The integrated analysis revealed a shared process of clinical judgment across both professional groups, while highlighting differences in how this process was enacted according to professional roles and responsibilities. In the present study, we refined the research question by integrating the two datasets and re-examining them using Tanner’s [5] Clinical Judgment Model and RTA [13]. This enabled us to explore extubation as a dynamic process rather than a discrete event and to illuminate aspects of clinical judgment that participants themselves found difficult to verbalise. In the previous studies, participants were recruited via consecutive sampling, and the datasets were analysed separately to capture the manifest content of the extubation experience from each professional perspective. Here, integrating the datasets enabled a deeper understanding of how CJ was enacted during extubation, making aspects of CJ visible that RNAs and ANs reported as difficult to articulate. This approach enabled us to integrate diverse viewpoints and evidence to develop a more comprehensive understanding of the phenomenon of extubation in the anaesthesia setting [14]. Although Tanner’s CJM was originally developed in nursing education, it provided a useful interpretive framework for understanding how clinicians described noticing, interpreting, responding, and reflecting during extubation. However, CJ during extubation also relies on embodied practical skills, situational awareness, tacit knowledge, and experiential competence developed through clinical practice and repeated exposure to complex situations. Previous research has highlighted the importance of practical experience, the development of situational awareness, and trust in one’s own competence in anaesthesia practice [17]. The model should therefore be understood as a supportive interpretive framework rather than a comprehensive representation of anaesthesia practice. Transferability of these findings should be considered in relation to the Swedish anaesthesia context, where RNAs often have a high degree of autonomy and extubation is commonly performed independently or in close collaboration with ANs. In other healthcare systems, professional responsibilities and decision-making authority during extubation may differ substantially. Such contextual differences may influence how CJ is enacted in practice. However, the core processes of noticing, interpreting, responding, and reflecting may remain relevant across settings in which extubation involves complex clinical decision-making.

The first author was not familiar with any of the participants prior to the previous studies. She provided information face-to-face and via e-mail to all previous study participants. They were informed that the studies aimed to gather insights into their extubation experiences and that the study results would be published in scientific journals. However, the fifth author (UN) was familiar with the RNAs in the focus groups at one hospital but acted only as an observer during the interviews. The first author, CM-J and UN were familiar with the data prior to this study. EB, BJL, and MH were not involved in data collection or analysis in earlier studies.

## Conclusion

Clinical judgment during extubation was identified as a dynamic and context-dependent process, enacted during a clinically vulnerable phase in which patients are at risk of complications and require close attention. In this context, RNAs and ANs continuously integrate clinical cues, experience, and situational awareness to ensure safe care.

Applying Tanner’s CJM provided a structured lens to illuminate this complexity, demonstrating how clinical practice is grounded not only in knowledge and technical skill, but also in experience, embodied intuition, and contextual sensitivity. CJ was thus understood as both an enacted process in the moment and a developmental process over time, where experience is continuously refined through reflection and carried forward into future practice.

Taken together, these findings highlight CJ in extubation as an evolving, experience-based practice in which knowing, acting, and reflecting are inseparably intertwined in the ongoing pursuit of patient safety.

### Implication for practice

The knowledge generated in this study on how RNAs and ANs express CJ during extubation is essential for informing clinical education and the training of future RNAs and ANs, particularly by supporting the development of anticipatory judgment, situational awareness, and reflective practice. It is crucial to highlight this critical moment for patient safety during anaesthesia and to encourage communication among anaesthesia professionals. The results of this study can also be used to provide support to new colleagues. Disruptions during extubation may interfere with focused attention and should therefore be considered in the organisation of clinical practice. Minimising unnecessary noise and disturbances in the OR is important during the vulnerable emergence from anaesthesia. Maintaining a calm environment around the patient and anaesthesia staff during extubation may support focused attention and safe care. To reduce the sense of isolation reported by RNAs, departments could introduce a brief pre-extubation team check that clarifies each team member’s role and expected behaviour during extubation. This study may inform the development of clinical guidelines that facilitate learning and competence development in extubation practice.

## Data Availability

The datasets generated and/or analysed during the current study are not publicly available due to confidentiality and ethical restrictions but are available from the corresponding author on reasonable request.
